# Attitudes of Healthcare Professionals and General Population Toward Vaccines and the Intention to Be Vaccinated Against COVID-19 in Spain

**DOI:** 10.3389/fpubh.2021.739003

**Published:** 2021-10-08

**Authors:** Isabel Iguacel, Aurelio Luna Maldonado, Aurelio Luna Ruiz-Cabello, Eva Samatán, Judith Alarcón, María Ángeles Orte, Silvia Santodomingo Mateos, Begoña Martínez-Jarreta

**Affiliations:** ^1^Department of Physiatry and Nursing, University of Zaragoza, Zaragoza, Spain; ^2^Instituto Agroalimentario de Aragón, Zaragoza, Spain; ^3^Instituto de Investigación Sanitaria Aragón, Zaragoza, Spain; ^4^Centro de Investigación Biomédica en Red de Fisiopatología de la Obesidad y Nutrición, Zaragoza, Spain; ^5^Health Sciences Department, University of Murcia, Murcia, Spain; ^6^Servicio Riojano de Salud, Logroño, Spain

**Keywords:** vaccines, COVID-19, attitudes, healthcare professionals, intention to vaccinate

## Abstract

**Background:** To achieve herd immunity, the acceptance of the COVID-19 vaccine by the population, especially healthcare professionals, plays a key role. The objective of the present paper is to address the differences in attitudes among Spanish healthcare professionals compared with the general population regarding COVID-19 vaccination.

**Methods:** This cross-sectional study included data from 2,136 adults (*n* = 664 healthcare professionals) from an online survey conducted from May 6 to June 9, 2021. The Vaccination attitudes examination scale was used to measure the negative attitudes toward vaccines. Four subscales: mistrust of vaccine benefit, worries about the unforeseen future effect, concerns about commercial profiteering, and preference for natural immunity were calculated. Generalized linear mixed models were conducted to study these associations.

**Results:** Between 10.2 and 22.6% of the subjects showed high levels of negative attitudes toward vaccines. However, only 1.5% of our sample (2.1% among healthcare professionals) refused to get the COVID-19 vaccine when it was offered because they chose otherwise. Retired people showed the lowest concerns and the highest trust in vaccines. No statistically significant effects were found between working in a healthcare field and having higher positive attitudes toward vaccines.

**Conclusion:** Low levels of rejection against the COVID-19 vaccine were identified in the present sample. However, despite being at a higher risk, health care professionals did not show higher positive attitudes toward vaccines. Furthermore, refusal percentage to vaccination was higher among healthcare professionals compared with non-healthcare professionals. Developing a strategy to increase positive attitudes against the COVID-19 vaccine should be an objective for public health policy.

## Introduction

In a global pandemic context, such as that generated by SARS-COV-2, immunization of the population through vaccination is a public health priority (1–3). The success of COVID-19 vaccination depends, to a large extent, on the confidence that the population has in vaccines. Different studies have shown the efficacy of the vaccines currently in the market, reporting levels above 90% for Pfizer, Moderna, and Sputnik V, or above 60% in the case of Vaxzevria (AstraZeneca) or Janssen (3). However, in recent months, confidence in vaccines (especially in the case of the Vaxzevria vaccine) has been dampened by doubts about their efficacy and reported side effects (4–7). Even though vaccines have undergone strict safety controls, it is also necessary that the information offered to the population and users be adequate and expressive of maximum transparency (8).

In a recent study carried out in England, 16% of respondents showed high levels of distrust about vaccines in one or more domains. Attitudes of distrust toward vaccination were higher among people from minority ethnic backgrounds, with lower levels of education, lower annual income, low awareness of COVID-19, and compliance with government COVID-19 guidelines. Overall, 14% of respondents reported being unwilling to receive a vaccine for COVID-19, while 23% were unsure (9).

Vaccine hesitancy can be considered a global health threat. But particularly is concerning the negative attitudes toward vaccination among healthcare professionals such as doctors, nurses, emergency medical personnel, dental professionals and students, medical and nursing students, laboratory technicians, pharmacists, hospital volunteers, and administrative staff. The acceptance of the COVID-19 vaccine among healthcare professionals plays a key role in combating the pandemic since they are not only among the first group to receive the vaccination but also, this group has an increased risk of contracting and transmitting disease and has a potentially powerful influence on patient vaccination decisions. Hence, it is important to consider their attitudes about COVID-19 vaccination to better address barriers to widespread vaccination acceptance. One of the possible solutions that Governments have proposed to increase COVID-19 vaccine rates is making this vaccine mandatory for several high-risk groups such as healthcare professionals, care home workers, or even all federal public servants and many other workers (10). A study conducted in Spain with data collected from September 10 to November 23, 2020, revealed that 22.43% of the respondents would not be vaccinated (of which 20–24% were non-health professionals or unemployed, 17.5% physicians, 31.5% other health professionals, and almost 35% nurses) (11).

At a time when cases of the Delta variant are rising sharply worldwide and countries are lifting restrictions, it is key to know the concerns and doubts that people have about vaccination. In a recent survey of 1,050 subjects conducted in Spain between March 17 and 18, most Spaniards (52%) responded that the Vaxzevria vaccine was unsafe, twice as many as a month ago (25%). However, the perception of the safety of the other three vaccines administered in Spain, Pfizer-BioNTech, Moderna, and Janssen, increased. The same attitude was recorded in Italy, France, and Germany (12). After the resumption of vaccination with Vaxzevria in Spain, official data showed that only 1% refused to receive it (12). However, there have been more consultations in primary care centers about the effects of the vaccines, although these seem to dissipate after the information provided by health professionals. Thus, it is important to provide as much transparency as possible, generating a climate of trust between government, healthcare professionals, and regular citizens to be vaccinated.

Although there are different ways to measure antivaccination attitudes (13), the current work includes a multidimensional conceptualization of antivaccination attitudes that encompasses four items derived from the vaccination attitudes examination (VAX) scale (14): (1) distrust about vaccine benefit, (2) concern about unpredictable vaccine effects in the future, (3) concerns about commercial profiteering, and (4) preference for natural immunity. The 12-item VAX scale is a short questionnaire with high internal consistency reliability (15).

Most of the investigations so far have focused on measuring attitudes toward vaccines rather than the explicit vaccination itself. Since intention does not always correlate with behavior o our purpose is to address differences in attitudes among Spanish healthcare professionals compared with the general population, including the actual acceptance level of the COVID-19 vaccine. Moreover, we aim to explore potential associations between sociodemographic factors (mainly gender, age, migrant status, education, and occupational status) and negative attitudes toward vaccines and intention to vaccinate in a sample of the Spanish population.

## Materials and Methods

### Study Design and Participants

Data were collected from an online anonymous survey conducted from May 6, 2021, to June 9, 2021. This cross-sectional study included a convenience sample of the adult population of Spain.

We used Google Forms, an online survey platform, to publish the questionnaire, and the link generated was then shared *via* social networks such as Facebook, Twitter, and WhatsApp or email. The interviewees visited the URL on their electronic devices to answer the questionnaire. Additionally, healthcare professionals who work at university hospitals of three medium-sized cities (Zaragoza, Logroño, and Murcia) were contacted *via* email with the support of the Health Research Institute of each city to get the maximum sample of this professional group. The inclusion criteria were individuals who (1) were 18 years old or older; (2) voluntarily agreed to participate in the online survey and (3) were able to read and complete the self-administered questionnaire independently.

After excluding those participants who did not complete more than 50% of the required questions (*n* = 45) a total of 2,136 were finally included in the present study. Since during data collection in Spain around 40% of the participants had not received any vaccination yet only 1,189 were asked to complete all the questions.

### Measures

#### Vaccine Attitudes and Intentions

The VAX scale was used to measure the negative attitudes toward vaccines (14). To answer this 12-item scale, participants were asked to focus on the specific COVID-19 vaccine they had received.

The VAX scale has been already adapted and translated into Spanish (16). In the present research, the VAX scale was translated from English to Spanish by author II (back-translation verified by another co-author JA) and finally revised by all co-authors who are experts in medicine and epidemiology. Internal consistency for the 12-item showed excellent internal consistency (Cronbach's α = 0.92). In agreement with our results, the VAX scale has been recently used in several investigations, showing a good validation of this scale in the context of the COVID-19 pandemic (9, 17, 18).

Responses were rated on a 6-point scale from 1 “strongly agree” to 6 “strongly disagree.”

Then, four sub-scales (1) mistrust of vaccine benefit (e.g., “I feel safe after being vaccinated.”), (2) worries about the unforeseen future effect (e.g., “I worry about the unknown effects of vaccines in the future”); (3) concerns about commercial profiteering (e.g., “Vaccines make a lot of money for pharmaceutical companies, but do not do much for regular people.”); and (4) preference for natural immunity (e.g., “Natural immunity lasts longer than a vaccination.”) (14), including three-items each respectively, were calculated. Therefore, a higher total score indicates more positive attitudes toward vaccinations, with three of the initial 12-items being reverse coded.

For descriptive purposes, the four subscales and the attitude toward vaccine question were grouped into high (a score of 5–6 on a scale of 1–6), intermediate (score of 3–4), and low (score of 1–2) levels of negative attitudes toward vaccines.

Similarly, the general attitude toward vaccines was assessed with the agreement on the currently recommended child and adolescent immunization schedule and recoded into the low agreement (score of 1–2 on a scale of 1–6), intermediate (score of 3–4), and high (score of 5–6).

All predictor variables were selected by reviewing the existing literature on the topic. Thus, we collected sociodemographic information such as gender (male vs. women), age group (18–25, 26–35, 35–45, 45–55, 56–65, and 65+), migrant status (born in Spain: yes vs. no), educational level (undergraduate; health-sciences-related graduate or postgraduate i.e., medicine, nursing, pharmacy; and non-health-sciences-related graduate or postgraduate), and occupation status (healthcare professional, non-healthcare professional, retired, and unemployed or student).

Moreover, we included some questions about receiving the flu vaccine in the previous year; the possibility of vaccination against COVID-19 (“no, for medical reasons,” –i.e., the hematologist recommended not to have it, “No, because I was pregnant,” “no, because they did not offer me the vaccine yet,” “no, because I just had COVID-19,” “no, because I refused to,” “yes, but I only got one dose (and I need two doses),” “yes, and I got all doses,” i.e., one for Janssen or two for Pfizer, Moderna or AstraZeneca/Vaxzevria; and the possibility of being vaccinated after knowing previously possible side effects “yes, of course,” “yes, but I would think about it more,” “I will have doubts,” “no, side effects that I had do not make up for it.”

### Statistical Analysis

For descriptive analyses, percentages and chi-square tests were used to evaluate the associations between sociodemographic characteristics such as gender, age, migrant status, education, and occupational status, and attitudes toward vaccination (agreement with current recommended Child and Adolescent Immunization Schedule, mistrust, worries about unforeseen effects, concerns about commercial profiteering and preference for natural immunity). Subsequently, we conducted generalized linear mixed models to study the associations between occupational status (healthcare professional, retired, unemployed or student, and non-healthcare professionals used as reference). For the above regression, betas and the respective 95% confidence intervals (CI) were estimated. Furthermore, since odds ratio (OR) can be more easily interpret for the reader, multinomial logistic regression models were also conducted after creating binary variables for each factor (four-subscales). As responses were rated on a six-point scale, the four subscales were grouped into a positive attitude (a score of 4–6 on a scale of 1–6) vs. a negative attitude (score of 1–3). All analyses were performed using SPSS version 26.0 (IBM Corporation, New York, NY, United States). The alpha level was set at 0.05, and *p* < 0.05 was considered statistically significant.

Raw models were unadjusted models and adjusted models included gender and sex. Since occupational status and education were highly correlated, educational status was not included as a confounding factor.

Finally, to compare the factor structure of each construct generated from the Spanish sample to the structures obtained in the original US samples, principal components analysis (PCA), was conducted for the VAX-scale used in the survey. PCA results with Varimax with Kaiser normalization to obtain a simple structure were reported in the results section (Supplementary Table 1).

## Results

Table 1 presents the characteristics of the sample of this study. About 33.1% of the sample were graduates or postgraduates in a Health Science field, and 31.1% had a healthcare occupation. Around 55.2% reported having been vaccinated against COVID-19 (27.6% with necessary doses, 44.8% were not vaccinated yet due to medical reasons (0.6%), pregnancy (0.8%), just had COVID-19 (0.5), or the vaccine had not been offered yet (38.6%). 1.5% of the sample affirmed that the vaccine had been offered but they had rejected the jab. This percentage (rejection of COVID-19 vaccine) was higher among healthcare professionals compared to non-healthcare professionals, students, or those unemployed. Almost half of the sample (47.3%) received a flu vaccine the previous year.

**Table 1:** Sample characteristics (*N* = 2,136).

***N*** **= 2,136**	***N***	**%**
**Age (in years)**		
18–25	307	14.4
26–35	223	10.4
36–45	526	24.6
46–55	480	22.5
56–65	369	17.3
>65	231	10.8
**Gender**		
Male	690	32.3
Female	1,446	67.7
**Born in Spain**		
Yes	1,977	92.6
No	136	6.4
**Education**		
Undergraduate	599	28.0
Graduate or Postgraduate (no Health Sciences related)	827	38.7
Graduate or Postgraduate (Health Sciences related)	710	33.2
**Occupation**		
Healthcare Professional	664	31.1
Non-healthcare Professional	986	46.2
Retired	157	7.4
Unemployed or Student	329	15.4
**Vaccination against COVID-19**		
No, for medical reasons	13	0.6
No, because I was pregnant	18	0.8
No, because they did not offer me the vaccine yet	825	38.6
No, because I just had COVID-19	59	2.8
No, because I refused to	32	1.5
Yes, but I only got one dose (and I need two doses)	590	27.6
Yes, and I got all doses	599	28.0
**Agreement with current recommended Child and**		
**Adolescent**		
Low	338	15.8
Moderate	172	13.9
High	1,607	75.2
Missing	19	0.9
**Flu vaccine in prior year**		
Received a flu vaccine	1,010	47.3
Did not receive a flu vaccine	1,113	52.1
Missing	13	0.6
***N*** **= 1.189 vaccinated with at least one dose**	***N***	**%**
**Possibility of being vaccinated after knowing side effects**		
Yes, of course	1,057	88.9
Yes, but I would think about it more	34	2.9
I will have doubts	32	2.7
No, side effects that I had do not make up for it	11	0.9
Missing	55	4.6
**Trust/Mistrust**		
Trust	873	73.4
Uncertain	94	4.4
High mistrust	217	10.2
Missing	5	0.4
**Worries about future effects**		
Low worries about unforeseen vaccine effects	513	24.0
Moderate worries about unforeseen vaccine effects	429	36.1
High worries about unforeseen vaccine effects	242	11.3
Missing	5	0.4
**Concerns about commercial profiteering**		
Low concerns	485	40.8
Moderate concerns	430	36.2
High concerns	269	22.6
Missing	5	0.4
**Preference for natural immunity**		
Low preference	426	19.9
Moderate preference	497	23.3
Strong preference	261	22.0
Missing	5	0.4

Around 10.2% of the sample manifested high mistrust about the safety of the vaccine, 11.3% expressed high worries about the unforeseen vaccine effects, 22.6% reported high concerns about commercial profiteering and 22.0% expressed strong preference.

Regarding the question of whether they would still be willing to receive the vaccine had they known the side effects they experienced, 88.9% of the sample had no doubts about being vaccinated. Nevertheless, 2.9% expressed that they would think about it more, 2.7% said to have some doubts and 0.9% had regrets due to the experienced side effects.

To investigate the validity of the VAX scale in Spanish and refute the existence of the four factors (the four abovementioned sub-scales), we conducted PCA. As shown in Supplementary Table 1 the number of extracted factors was four and they accounted for 79.79% of the explained variance. The Varimax rotated solution for the VAX-scale was presented.

Table 2 shows attitudes toward vaccines (agreement with current recommended Child Immunization Schedule and the four domains of the VAX scale trust/mistrust, worries about unforeseen effects, concerns about commercial profiteering, preference for natural immunity) by gender, age, migrant origin, education status, and occupation (in percentage).

**Table 2:** Attitudes toward vaccines (agreement with current recommended Child Immunization Schedule, trust/mistrust, worries about unforeseen effects, concerns about commercial profiteering, preference for natural immunity) by gender, age, migrant origin, education status and occupation (in percentage).

	**Agreement with recommended**			**Trust/Mistrust**			**Worries unforeseen**			**Concerns commercial**			**Preference for**		
	**Child immunization schedule**						**effects**			**profiteering**			**natural immunity**		
**In percentage (%)**	**Low**	**Moderate**	**High**	**Trust**	**Uncertain**	**High mistrust**	**Low**	**Moderate**	**High**	**Low**	**Moderate**	**Strong**	**Low**	**Moderate**	**High**
**Gender**															
Male	15.1	8.2	76.7	79.0	5.6	14.8	45.3	31.1	23.1	43.2	37.3	18.9	34.9	40.2	24.3
Female	16.4	8.1	75.6	71.2	8.8	19.6	42.3	38.1	19.3	39.8	35.7	24.1	36.2	42.4	21.0
*p*-value	0.267			**0.036**			0.120			0.261			0.601		
**Age (in years)**															
18–25	23.0	5.6	71.4	73.8	3.3	22.4	27.6	45.2	26.7	46.7	34.3	18.6	42.9	38.6	18.1
26–35	17.0	13.0	70.0	69.8	7.3	22.9	36.5	39.6	24.0	39.6	40.6	19.8	47.9	36.5	15.6
36–45	16.4	9.8	73.8	69.1	9.9	20.9	45.5	33.0	21.5	38.2	37.2	24.6	35.1	41.9	23.0
46–55	14.1	8.6	77.3	75.3	6.9	17.8	47.2	33.3	19.5	41.4	35.6	23.0	35.6	45.4	19.0
56–65	13.3	6.0	80.7	75.4	8.1	15.8	46.5	36.7	16.2	43.4	34.0	21.9	33.0	42.4	23.9
>65	12.5	5.4	82.1	74.2	11.3	13.6	51.1	29.9	18.1	33.9	38.5	26.7	28.5	43.4	27.1
*p*-value	**0.008**			0.104			**0.001**			0.508			0.073		
**Born in Spain**															
No	14.1	7.6	69.6	66.0	14.9	18.4	42.6	34.0	23.4	36.2	38.3	25.5	29.8	42.6	27.7
Yes	16.1	16.3	76.4	73.6	7.5	19.1	42.9	36.2	20.5	41.1	35.8	22.6	36.1	41.5	21.9
*p*-value	0.120			0.480			0.470			0.832			0.634		
**Education**															
Undergraduate	15.4	8.1	76.5	75.4	8.5	15.9	46.5	35.4	17.8	34.6	41.1	24.1	33.7	44.8	21.2
Graduate or Postgraduate (no Health Sciences related)	16.8	10.3	72.9	71.2	9.7	18.7	47.9	32.7	19.1	38.5	37.0	24.1	35.0	42.4	22.2
Graduate or Postgraduate (Health Sciences related)	15.5	5.6	78.8	73.2	6.7	19.5	39.0	38.0	22.5	45.6	32.8	21.1	37.5	39.7	22.3
*p*-value	0.942			0.606			0.186			**0.049**			0.846		
**Occupation**															
Healthcare professional	16.8	4.0	79.2	74.1	6.7	18.8	44.1	35.2	20.3	42.2	34.6	22.8	35.2	41.6	22.8
Retired	11.1	4.1	84.8	74.8	8.6	15.2	50.3	28.5	19.9	31.1	37.7	29.8	27.8	43.0	27.8
Unemployed or Student	19.3	3.9	76.8	74.2	6.6	19.2	30.6	44.1	25.3	46.3	35.4	18.3	43.7	38.9	17.5
Non-healthcare professional	16.1	5.7	78.2	70.7	11.0	18.0	47.7	28.5	19.9	38.9	38.9	21.9	35.0	43.8	20.8
*p*-value	0.426			0.326			**0.001**			0.066			0.061		

*In bold significant results of Pearson Chi-Square*.

By gender, women showed a statistically significantly higher percentage of mistrust (19.6%) compared to men (14.8%). By age, younger adults (18–25 years old) presented a higher percentage of 1) lower agreement with recommended child and immunization schedule (23%) and 2) worries about unforeseen effects (26.7%) compared with elderly (12.5 and 18.1%, respectively. Those who were not born in Spain had a statistically significantly higher percentage of lower agreement with the recommended child immunization schedule (16.1 compared with 14.1% of those born in Spain.

By education, those who were graduates or postgraduates in a field related to health sciences showed a higher agreement with the current recommended child immunization schedule (78.8%) and lower concerns about commercial profiteering (21.1%) compared with those with studies in a different field (72.9, 24.1%, respectively).

By occupation, healthcare professionals had a higher percentage of agreement with the current recommended Child Immunization Schedule (78.2%), a lower percentage of worries about unforeseen effects (44.1), and lower concerns of commercial profiteering (42.2%) compared with non-healthcare professionals (73.1, 47.7, and 38.9%, respectively).

The lowest level of negative attitudes (or the highest level of positive attitudes toward vaccines) was shown by retired people compared with unemployed/students, healthcare professionals, or non-healthcare professionals.

Supplementary Table 2 indicates the intention to vaccinate by occupation status in percentage. The present crosstab shows that most of the healthcare professionals in Spain were already vaccinated with all necessary doses (64%). Besides, 2.1% of the healthcare professionals reported not to have been vaccinated because they refused to, compared to unemployed or students (0.3%), non-healthcare professionals (1.3%), or retired (2.5%).

Results from Generalized Linear Mixed Models to study the associations be between agreement with current Child Immunization Schedule and occupational status are presented in Table 3. Models (unadjusted and unadjusted) showed a statistically significant higher agreement with the current Child Immunization Schedule and occupational from retired people (beta =-0.5; 95% CI =-0.99–0.16) compared to non-healthcare professionals.

**Table 3:** Associations between agreement with current Child Immunization Schedule and occupational status. Results from Generalized Linear Mixed Models.

	**Unadjusted model**		**Adjusted model**	
	**β**	**95% CI**	**β**	**95% CI**
**Occupation**				
**Agreement with recommended Child Immunization Schedule**				
Healthcare professional	−0.10	−0.27–0.07	−0.09	−0.27–0.08
Retired	**−0.49**	**−0.78−0.19**	**−0.50**	**−0.99−0.16**
Unemployed or Student	−0.01	−0.23−0.21	−0.26	−0.55–0.02
Non-healthcare professionals	Ref.		Ref.	

*Statistically significant results are shown in bold font. Adjusted models were adjusted for age and sex*.

Finally, Table 4 presents results from Generalized Linear Mixed Models to study the associations between occupational status and negative attitudes toward vaccines (mistrust, worries about unforeseen effects, concerns about commercial profiteering, and preference for natural immunity). Those who were unemployed, or students presented higher worries about unforeseen effects of vaccines compared to non-healthcare professionals (beta = 0.39; 95% CI = 0.17–0.62). After adjusting for age and sex this relationship was no longer significant (beta = 0.23; 95% CI = −0.08–0.53). No other statistically significant effects were detected. Similar results were found when conducting multinomial logistic regression models (Supplementary Table 3).

**Table 4:** Associations between occupational status and negative attitudes toward vaccines (mistrust, worries about unforeseen effects, concerns about commercial profiteering, and preference for natural immunity). Results from Generalized Linear Mixed Models.

	**Mistrust**		**Mistrust**		**Worries unforeseen**		**Worries unforeseen**		**Concerns commercial**		**Concerns commercial**		**Preference for natural**		**Preference for natural**	
	**(unadjusted model)**		**(adjusted model)**		**effects (unadjusted**		**effects (adjusted**		**profiteering**		**profiteering**		**immunity**		**immunity**	
					**model)**		**model)**		**(unadjusted model)**		**(adjusted model)**		**(unadjusted model)**		**(adjusted model)**	
	**β**	**95% CI**	**β**	**95% CI**	**β**	**95% CI**	**β**	**95% CI**	**β**	**95% CI**	**β**	**95% CI**	**β**	**95% CI**	**β**	**95% CI**
**Occupation**																
Healthcare professional	0.00	−0.22–0.23	−0.11	−0.35–0.13	0.09	−0.1–0.27	0.06	−0.14–0.25	0.06	−0.17–0.29	0.10	−0.14–0.34	−0.08	−0.30–0.14	−0.16	−0.40–0.07
Retired	−0.15	−0.47–0.16	−0.06	−0.52–0.39	−0.13	−0.39–0.12	−0.13	−0.51–0.23	0.41	−0.73–−0.10	−0.41	−0.87–0.06	−0.26	−0.56–0.04	−0.11	−0.55–0.33
Unemployed or Student	0.76	−0.20–0.35	−0.06	−0.44–0.32	**0.39**	**0.17–0.62**	0.23	−0.08–0.53	0.27	−0.01–0.55	0.29	−0.10–0.68	0.24	−0.02–0.51	0.15	−0.22–0.51
Non-healthcare professionals	Ref.		Ref.		Ref.		Ref.		Ref.		Ref.		Ref.		Ref.	

*Statistically significant results are shown in bold font. Models Adjusted models were adjusted for age and sex*.

## Discussion

The present study aimed to investigate the attitudes and intention to vaccinate in a sample of 2,136 adults in Spain. In this cross-sectional analysis, we collected information from May 6 to June 9, 2021, to identify characteristics associated with more negative attitudes and lower intention to vaccinate by occupational status.

In this study, despite a substantial percentage of negative attitudes (above 10% of high mistrust, high worries about unforeseen vaccine effects, and above 20% of high concerns about commercial profiteering and the strong preference for natural immunity), only 1.5% of the total sample claimed they had not been vaccinated because they had refused to do it. Additionally, another 1.4% refused to get the vaccination of which 0.6% was due to pregnancy and 0.8% for medical reasons (i.e., due to my conditions my medical doctor recommended not to get vaccinated).

Nevertheless, contrary to our expectations this percentage (rejection of COVID-19 vaccine) was higher among healthcare professionals (2.1%) and retired people (2.5%) compared with non-healthcare professionals (1.3%), students, or those unemployed (0.3%). These results suggest differences between attitudes and behaviors. Although these results should be more investigated in further studies, we can hypothesize that due to a higher perceived pressure to get vaccinated among healthcare professionals and elderly people these groups might be more likely to reject the COVID-19 vaccine (19).

Also, we have to take into account that there are fluctuations in attitude and also in behavior that have been observed in the Spanish population and other countries and therefore in health professionals (20).

Moreover, when asked whether they would still be willing to receive the vaccine had they known the side effects they experienced, around 90% of the sample had no doubts about being vaccinated. However, 3.6% had some doubts or regrets due to the experienced side effects that did not make up for it. On the one hand when studying the negative attitudes toward vaccines between healthcare professionals and non-healthcare professionals no statistically significant results were found in multinomial linear regressions. A systematic review collecting data until February 2021 found that vaccine acceptance among healthcare professionals varied widely and ranged from 27.7 to 77.3%. Although healthcare professionals had positive attitudes toward future COVID-19 vaccines, vaccine hesitancy was still common, particularly among nurses (21). Concerns for safety, efficacy and effectiveness, and distrust of the government were the most important barriers cited. Compared to nurses, physicians have a higher level of confidence in vaccines. These results can be explained by the fact that nurses have a lower degree of medical training as well and higher contact with patients, which contributed to lower uptake of vaccines. In our study, we did not ask participants to specify the profession, which can be explained why we did not find differences between healthcare and non-health care professionals.

In a survey conducted in Israel, significant differences regarding the intention to vaccinate between physicians, nurses, and the general population were found. A lower acceptance was obtained in nurses (61%) compared with the general population (75%) and doctors (78%) (22). These results are in line with a study conducted in the United States, where nurses and patient care associates were among those with the least intended to be vaccinated in comparison to medical students and physicians who were the highest (23).

Same results were also found in other countries of Europe, such as Belgium, as well as in Asia, such as Hong Kong, where again nurses were unsure about taking the vaccine while physicians had a more positive attitude (24–26).

A deeper knowledge of the vaccine may be the key to achieve higher positive attitudes and behaviors and it can be the cause of the differences found among doctors, nurses, and other health professionals.

Generally, the eligibility criterion used to prioritize vaccination was to start by vaccinating those most exposed, that is, healthcare professionals in contact with patients, and people working in nursing homes (27). Since these healthcare professionals (mainly doctors, nurses, and those professionals working at nursing home residences) have been among the first vaccinated in Spain and can exert an impact on getting people vaccinated, it is essential to increase the positive attitudes among healthcare professionals.

On the other hand, retired people (who are generally people above 65 years old in Spain) had the lowest concerns in vaccines and the highest trust.

Previous studies in Spain observed a low acceptance of the vaccine against COVID-19 in the phase prior to its availability (11, 28). In agreement with a previous study, our results demonstrated that in spite of initial suspicions, Spain has one of the highest acceptance rates worldwide (29).

Along with other studies, women, younger adults (<35 years old), those who were not born in Spain, those who study a degree in a field different to Health Sciences, and those who were unemployed or students presented a higher percentage of negative attitudes toward vaccines compared with men, elderly, native Spanish people, those who study a degree in a field related to Health Sciences and those retired (30, 31).

According to the latest data for Spain (June 22, 2021), there are 50.8% of people received at least one dose of the COVID-19 vaccine (32). Although our data has shown low levels of COVID-19 vaccine rejection in Spain (1.5%), vaccine hesitancy among healthcare professionals is specially concerning.

In view of these results, it seems that knowledge and perceived risk in different age groups or exposure/profession can be determinants in terms of attitude and behaviors. Nonetheless, we have not studied it in detail, and this could be a future line of research.

To our knowledge, this is the first study conducted in Spain to investigate the attitudes toward vaccines and intention to vaccinate against COVID-19 by occupational status and other socioeconomic characteristics with a sample size of 2,136 individuals (of which 31.1% were healthcare professionals) after the approval of the vaccine. In addition, although we used convenience sampling, we have a good representation of different age groups, and women represented 67.7% of the respondents, which is in agreement with the percentages of healthcare professionals in Spain and worldwide (33). Besides, our sample included information from three different cities of medium size in Spain (Zaragoza, Logroño, and Murcia). These cities (specially Zaragoza) are frequently chosen to carry out different studies because the sociodemographic profile and level of income of the sample were representatives of the Spanish population. Hence, the results of the present study could be extrapolated to the whole country (34, 35).

Nevertheless, our investigation has several limitations. This study is not random and therefore is not representative of the Spanish population. Moreover, there are some groups that could be underestimated, in part due to the collection method used, (i.e., men represented 32.3% of the sample or migrants were 6.4%). These differences among groups may affect the results of our study (men and natives appear to have higher positive attitudes compared with women and migrants, respectively). Therefore, the extrapolation of these results can be difficult. Although online questionnaires are simple tools that can offer advantages such as access to different populations and prompt answers, some questions that can arise when auto-filling the questionnaire and could be responded in a face-to-face interview are difficult to address online surveys. Finally, we did not differentiate between the various healthcare professionals, and consequently, future line investigations should discriminate between doctors, nurses, auxiliary nurses, pharmaceutical industry professionals, administrative staff, and also those who are on the front line against COVID-19 and those who are not. Consequently, results should be interpreted and considered based on all that.

## Conclusions

A considerable percentage (between 10.2 and 22.6%) of the present sample showed high levels of negative attitudes toward vaccines (high mistrust, worries about unforeseen effects, concerns about commercial profiteering, and preferences for natural immunity). However, only 1.5% of the sample (2.1% among healthcare professionals) refused to get a COVID-19 vaccine when it was offered because they chose otherwise.

Notwithstanding, healthcare professionals can be at an increased risk of being infected with COVID-19, and no statistically significant effects were found between working in the healthcare field and higher positive attitudes toward vaccines. Developing a strategy to increase positive attitudes against the COVID-19 vaccine should be an objective for public health policy.

## Data Availability Statement

The raw data supporting the conclusions of this article will be made available by the authors, without undue reservation.

## Ethics Statement

The studies involving human participants were reviewed and approved by Comité de Ética de la Investigación de la Comunidad Autónoma de Aragón (CEICA). Affiliation: Instituto Aragonés de Ciencias de la Salud (IACS). The patients/participants provided their written informed consent to participate in this study.

## Author Contributions

II analyzed the data and drafted the manuscript. BM-J and ES critically revised the manuscript for important intellectual content. All authors contributed to the design of the study, approved the final version, and take responsibility for the content.

## Conflict of Interest

The authors declare that the research was conducted in the absence of any commercial or financial relationships that could be construed as a potential conflict of interest.

## Publisher's Note

All claims expressed in this article are solely those of the authors and do not necessarily represent those of their affiliated organizations, or those of the publisher, the editors and the reviewers. Any product that may be evaluated in this article, or claim that may be made by its manufacturer, is not guaranteed or endorsed by the publisher.
