# Supplementary material for: Attitudes of Healthcare Professionals and General Population Toward Vaccines and the Intention to Be Vaccinated Against COVID-19 in Spain
Source: Front Public Health. 2021 Oct 8;9:739003. doi: 10.3389/fpubh.2021.739003 (PMC8531478; doi:10.3389/fpubh.2021.739003)
Supplement: Supplementary file 1 [file Data_Sheet_1.PDF]

SUPPLEMENTARY MATERIAL

Table S1. VAX scale items rotated component matrix. Each component represents a factor, and each item represents a question in the survey.

**Items**	Mistrust of vaccine benefit	Worries about unforeseen future effects	Concerns about commercial profiteering	Preference for natural immunity
I feel safe after being vaccinated (–)	.944			
I can rely on vaccines to stop serious infectious diseases (–)	.902			
I feel protected after getting vaccinated (–)	.945			
Although most vaccines appear to be safe, there may be problems that we have not yet discovered.		.674		
Vaccines can cause unforeseen problems in		.833		

children.				
I worry about the unknown effects of vaccines in the future.		.733		
Vaccines make a lot of money for pharmaceutical companies, but do not do much for regular people.			.572	
Authorities promote vaccination for financial gain, not for people's health.			.901	
Vaccination programs are a big con			.911	
Natural immunity lasts longer than a vaccination.				.808
Natural exposure to viruses and germs gives the safest protection.				.870
Being exposed to diseases naturally is safer for the immune system than being exposed through vaccination				.766

Note: Extraction Method: Principal Component Analysis. Rotation Method: Varimax with Kaiser Normalization

Table S2: Intention to vaccinate by occupation status (in percentage).

	No, for medical reasons	No, because they did not offer me the vaccine yet	No, because I was pregnant	No, because I just had COVID-19 (<6 months ago)	No, because I refused to	Yes, but I only got one dose (and I need two doses)	Yes, and I got all necessary doses
**Occupation (%)**	*						
Healthcare professional	0.6	12.7	2.0	3.5	2.1	15.4	64.0
Retired	0.0	0.0	0.0	1.3	2.5	40.1	56.1
Unemployed or Student	0.9	26.7	0.3	2.1	0.3	63.2	6.4
Non-healthcare professional	0.6	66.2	0.4	2.7	1.3	22.0	6.7

*Significant results of Pearson Chi-Square

Table S3: Associations between occupational status and negative attitudes towards vaccines (mistrust, worries about unforeseen effects, concerns about commercial profiteering and preference for natural immunity). Results from Multinomial Logistic Regressions.

	**Mistrust** (adjusted model)		**Worries unforeseen effects** (adjusted model)		**Concerns commercial profiteering** (adjusted model)		**Preference for natural immunity** (adjusted model)	
**Occupation**	**OR**	**95% CI**	**OR**	**95% CI**	**OR**	**95% CI**	**OR**	**95% CI**
Healthcare professional	1.06	0.69-1.63	0.92	0.62-1.38	1.06	0.79-1.42	1.30	0.92-1.80
Retired	1.12	0.48-2.64	1.10	0.52-2.33	1.31	0.75-2.67	0.96	0.51-1.81
Unemployed or Student	0.82	0.41-1.64	0.56	0.30-1.03	0.83	0.52-1.34	1.09	0.64-1.87
Non-healthcare professionals	Ref.		Ref.		Ref.		Ref.	

Models Adjusted models were adjusted for age and sex.
